# Synergistic Effect of Sugarcane Bagasse and Zinc Oxide Nanoparticles on Eco-Remediation of Cadmium-Contaminated Saline Soils in Wheat Cultivation

**DOI:** 10.3390/plants14010085

**Published:** 2024-12-30

**Authors:** Emad M. Hafez, Khadiga Alharbi, Hany S. Gharib, Alaa El-Dein Omara, Essam Elatafi, Maha M. Hamada, Emadelden Rashwan, Tarek Alshaal

**Affiliations:** 1 Department of Agronomy, Faculty of Agriculture, Kafrelsheikh University, Kafr El-Sheikh 33516, Egypt; emadhafez2014@gmail.com; 2 Institute of Agricultural Resources and Environment, Jiangsu Academy of Agricultural Sciences, Nanjing 210014, China; 3 Department of Biology, College of science, Princess Nourah bint Abdulrahman University, P.O. Box 84428, Riyadh 11671, Saudi Arabia; kralharbi@pnu.edu.sa; 4 Department of Microbiology, Soils, Water Environment Research Institute, Agricultural Research Center, Giza 12112, Egypt; alaa.omara@yahoo.com; 5 Department of Pomology, Faculty of Agriculture, Mansoura University, Mansoura 35516, Egypt; eelatafi@mans.edu.eg; 6 Agronomy Department, Faculty of Agriculture, Ain Shams University, Cairo 11566, Egypt; maha_hamada@agr.asu.edu.eg; 7 Agronomy Department, Faculty of Agriculture, Tanta University, Tanta 31527, Egypt; emad.rashwan@agr.tanta.edu.eg; 8 Department of Applied Plant Biology, Faculty of Agricultural and Food Sciences and Environmental Management, University of Debrecen, Böszörményi Str. 138, 4032 Debrecen, Hungary; 9 Soil and Water Department, Faculty of Agriculture, University of Kafrelsheikh, Kafr El-Sheikh 33516, Egypt

**Keywords:** sugarcane bagasse, zinc oxide nanoparticles, cadmium contamination, soil salinity, wheat productivity, antioxidant enzymes

## Abstract

Soil contamination with cadmium (Cd) and salinity poses a significant challenge, affecting crop health and productivity. This study explores the combined application of sugarcane bagasse (SCB) and zinc oxide nanoparticles (ZnO NPs) to mitigate the toxic effects of Cd and salinity in wheat plants. Field experiments conducted in Cd-contaminated saline soils revealed that the application of SCB (0, 5, and 10 t ha^−1^) and ZnO NPs (0, 12.5, and 25 mg L^−1^) significantly improved key soil physicochemical properties, including soil pH, electrical conductivity (EC), and exchangeable sodium percentage (ESP). The combined application of SCB and ZnO NPs significantly mitigated the effects of Cd and salinity on soil and wheat plants. SCB (10 t ha^−1^) reduced soil pH by 6.2% and ESP by 30.8% compared to the control, while increasing microbial biomass by 151.1%. ZnO NPs (25 mg L^−1^) reduced Cd accumulation in wheat shoots by 43.3% and seeds by 46.3%, while SCB and ZnO NPs combined achieved a reduction of 74.1% and 62.9%, respectively. These amendments enhanced antioxidant enzyme activity, with catalase (CAT) increasing by 35.3% and ascorbate peroxidase (APX) by 54.9%. Wheat grain yield increased by 42% with SCB alone and by 75.2% with combined SCB and ZnO NP treatment, underscoring their potential as eco-friendly soil amendments for saline, Cd-contaminated soils. These results underscore the potential of SCB and ZnO NPs as eco-friendly amendments for improving wheat productivity in contaminated soils, offering a promising strategy for sustainable agriculture in salt-affected areas.

## 1. Introduction

The contamination of agricultural soils with heavy metals, such as cadmium (Cd), has become a significant global challenge due to its escalating environmental impact [1]. Cd not only degrades soil quality and harms plant health but also poses serious risks to human and animal health, as it enters the food chain and accumulates in various plant tissues [2]. In Egypt, Cd-contaminated soils are widespread in the northern and central Delta regions, where agricultural lands are irrigated with water from the Kitchener Drain, which is heavily polluted with metals like Cd, Pb, and Ni [3]. The analysis of water and soil samples shows that Cd is the most abundant of these pollutants, and the Kitchener Drain remains the primary source of irrigation for the region’s growing agricultural areas [4]. The drain’s discharge into the Mediterranean Sea and Nile River further threatens marine ecosystems and drinking water supplies [5]. While treatment technologies for irrigation water are essential, they are often beyond the capacity of many countries. As a result, a global strategy has emerged to find sustainable solutions that mitigate the risks of using wastewater for agriculture, while preserving soil quality and plant health [6]. Additionally, salinized soils are widespread in tropical and subtropical regions, including North Africa and Egypt [7]. This salinization deteriorates soil structure and chemical properties, reduces microbial activity, and threatens crop growth and productivity, ultimately jeopardizing global food security [8].

Wheat is one of the most important strategic cereal crops worldwide, particularly in Egypt [9]. It serves as a staple food source for producing bread, flour, pastries, and sweets, while its residues are used as straw for livestock feed [10]. Wheat occupies the largest cultivated area in Egypt, and the government is actively working to expand its cultivation in order to reduce the gap between production and consumption [11]. However, wheat farming faces significant challenges due to poor soil quality caused by salinity and deteriorating irrigation water quality, primarily due to heavy metal contamination, especially Cd [12]. As a result, researchers are increasingly focusing on finding sustainable, eco-friendly solutions derived from organic sources to mitigate the risks posed by Cd pollution in both soil and irrigation water, with the goal of improving soil health and plant quality [13,14].

A modern approach to mitigating the harmful effects of soil salinity and Cd contamination is the use of soil amendments [15]. While previous studies have explored various amendments, biochar derived from sugarcane bagasse (SCB biochar) has proven to be particularly effective as a pollutant barrier. It significantly improves soil quality, properties, and the microbial community, thereby reducing Cd and Na uptake and their accumulation in plant aerial parts [16]. SCB is a lignocellulosic biomass composed of cellulose (48%), hemicellulose (28%), lignin (22%), and extractives (2%). It also contains high levels of silica (60%) and organic matter (38%) [17]. With a low ash content of around 4%, SCB has a comparative advantage over other plant residues used to produce biochar [18]. As a byproduct of the sugarcane processing industry, approximately 2.8 million tons of SCB are produced annually [19]. SCB undergoes slow thermal decomposition under anoxic conditions at 500 °C to produce biochar, which has been shown to immobilize heavy metals, such as Ni and Cd, in contaminated soils [20]. Furthermore, biochar from plant residues has been found to enhance soil organic matter and nutrient availability, particularly in saline soils [21,22].

Recent studies have confirmed that biochar alone is insufficient to fully prevent the transport of Cd or Na within plants or to maintain plant health without the addition of organic components that complement biochar’s vital role [23]. In this context, nanotechnology has emerged as a promising approach to mitigate abiotic stresses, such as heavy metal contamination and soil salinity, while also improving agricultural productivity [24]. Nanoparticles (NPs), with sizes smaller than 100 nm, exhibit unique physical and chemical properties, including a larger surface area, enhanced reactivity, and stronger magnetic properties compared to traditional plant growth regulators. These characteristics enable NPs to be absorbed rapidly through plant stomata when sprayed, with minimal loss [25]. NPs are considered safe, environmentally friendly, and nontoxic at small doses, and they can be derived either from natural sources (biosynthesis) or through chemical and physical methods (synthesis) [26]. Zinc, typically found in soils at concentrations of 1–10 parts per million, originates from rocks [27]. In alkaline soils, zinc becomes less available to plants and must be applied as a foliar spray to ensure proper absorption [28]. While Zn is more readily available in acidic soils (pH below 6), it remains less accessible in alkaline soils, requiring Zn fertilizers to be sprayed directly onto leaves for effective uptake [29]. Although exogenous Zn mixed with amino acids, sugar alcohols, or citric acid has been used as a foliar spray, it is poorly soluble in water [17]. Zinc oxide nanoparticles (ZnO NPs) have become one of the most commonly used nanocomposites in agriculture, demonstrating significant impacts on improving plant growth and resistance to abiotic stresses, like soil salinity and heavy metal pollution. ZnO NPs have shown superior performance compared to traditional Zn forms, such as Zn salts [30]. Recent studies have highlighted the ability of ZnO NPs to enhance the absorption of essential nutrients, while suppressing harmful elements, like heavy metals and Na ions, due to their bioavailability. However, further research is needed to investigate the potential of ZnO NPs in mitigating the combined damage caused by Cd contamination and soil salinity in wheat plants [31].

In light of the above, this study aims to investigate the soil–plant response to saline soil contaminated with Cd by using SCB and ZnO NPs as an effective remediation strategy. The focus is on their role in mitigating the effects of contamination to improve soil quality and food safety. The research will evaluate the combined treatment, applied both to the soil and as a foliar spray, to (1) examine the BCF, translocation factor, and bioaccumulation coefficient of Cd from the soil to different parts of the wheat plant; (2) assess the effects on soil physiochemical properties, enzyme activity, and microbial activity; and (3) analyze the physiological and biochemical changes in the plant, as well as the expression levels of CAT, APX, and Mn-SOD genes, in relation to yield.

## 2. Results

### 2.1. Physical and Chemical Properties of the Soil

Although Cd-contaminated saline soils negatively impacted soil physicochemical properties (e.g., pH, EC, and ESP) and microbial parameters (e.g., soil CO_2_ and microbial biomass), the use of soil amendments—either SCB, ZnO nanoparticles (ZnO NPs) via foliar application, or their combination—significantly improved these properties (Table 1). SCB alone was more effective than ZnO NPs in enhancing soil physicochemical and microbiological parameters. Specifically, applying SCB at 5 and 10 tons per hectare reduced pH (2.95–6.22%), EC (11.76–23.39%), and ESP (18.16–30.83%) compared to the control. SCB also increased CO_2_ influx (55.40–123.41%) and microbial biomass (77.77–151.07%) at the same concentrations. In contrast, foliar application of ZnO NPs (12.5 and 25 mg L^−1^) only reduced ESP slightly (by 0.15% and 25 mg L^−1^, respectively) and increased CO_2_ influx (7.06–5.58%) and microbial biomass (6.54–5.52%) compared to the control.

**Figure 1:**
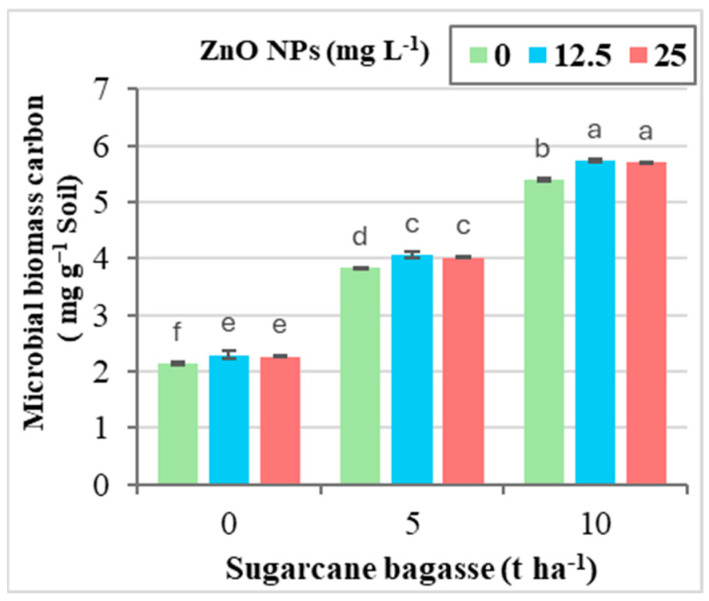
The interaction effect between sugarcane bagasse (SCB) (t ha^−1^) and zinc oxide nanoparticles (ZnO NPs) (mg L^−1^) on microbial biomass content (mg g^−1^ soil). Different letters on bars show significant differences at the level of *p* ≤ 0.0.5 according to Tukey’s test. Data are mean ± SD.

As shown in Figure 1, the combined application of SCB at 10 tons ha^−1^ and ZnO NPs at 12.5 mg L^−1^ had the greatest impact on enhancing microbial biomass (168.64% increase). However, the interaction between SCB and ZnO NPs did not significantly affect soil physicochemical properties, except for microbial biomass content, where a clear positive interaction was observed.

### 2.2. Soil Enzymes and Bacteriological Activity

**Figure 2:**
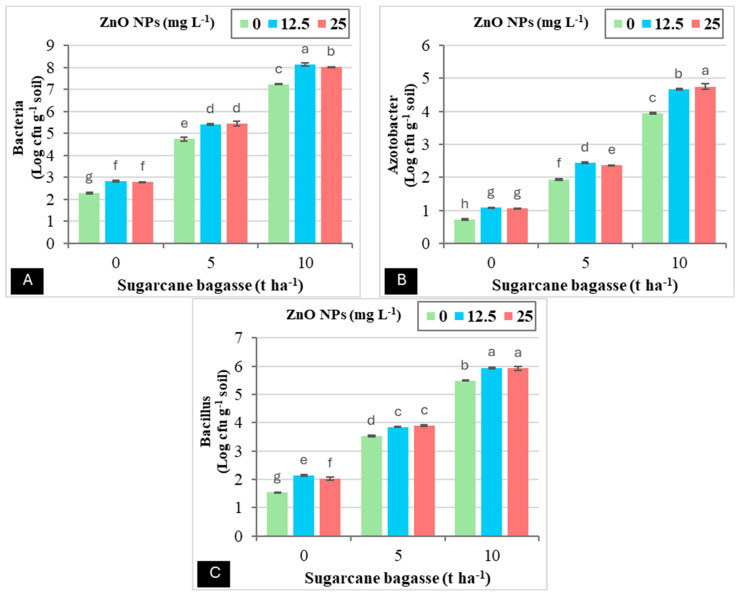
The interaction effect between sugarcane bagasse (SCB) (t ha^−1^) and zinc oxide nanoparticles (ZnO NPs) (mg L^−1^) on (**A**), bacteria (Log cfu g^−1^ soil) (**B**), *Azotobacter* (Log cfu g^−1^ soil) (**C**), and *Bacillus* (Log cfu g^−1^ soil) (**D**). Different letters on bars show significant differences at the level of *p* ≤ 0.0.5 according to Tukey’s test. Data are mean ± SD.

Using singular soil amendments, including SCB and the foliar applications of ZnO NPs or their combination, significantly enhanced soil enzyme activity and microbiological activity. The results presented in (Table 2) illustrate that the application of SCB alone markedly increased the activity of soil enzymes, specifically dehydrogenase and alkaline phosphatase, as well as the microbiological activity of bacteria, *Azotobacter*, and *Bacillus*, in comparison to the foliar application of ZnO NPs. When comparing treatments involving SCB to the control (without SCB), the application of SCB at rates of 5 and 10 tons per hectare resulted in significant increases in various metrics: dehydrogenase activity increased by 142.42–258.13%, alkaline phosphatase by 83.67–170.91%, bacterial counts by 97.09–195.41%, *Azotobacter* by 135.97–367.17%, and *Bacillus* by 97.26–203.68%. It was observed that a noteworthy interaction occurred between the co-application of SCB (10 tons ha^−1^) and ZnO NPs (12.5 mg L^−1^), which led to increases in bacterial populations (254.28%) and *Bacillus* (285.71%) compared to the control treatment. Furthermore, as seen in Figure 2, the combination of SCB (10 tons ha^−1^) and ZnO NPs (25 mg L^−1^) resulted in a significantly greater increase in *Azotobacter* (554.59%) relative to the control.

### 2.3. Cd Accumulation in Soil and Different Plant Parts

**Figure 3:**
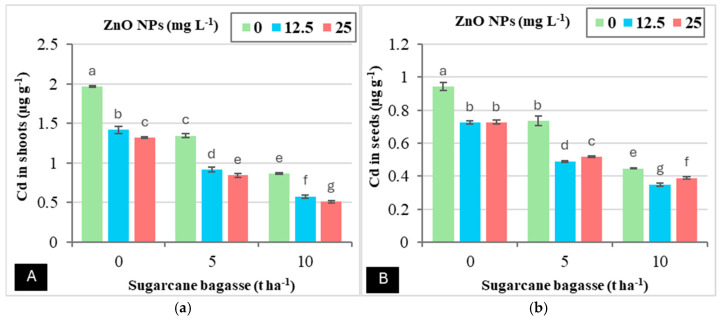
The interaction effect between sugarcane bagasse (SCB) (t ha^−1^) and zinc oxide nanoparticles (ZnO NPs) (mg L^−1^) on shoot cadmium (**A**) and seed cadmium (**B**). Different letters on bars show significant differences at the level of *p* ≤ 0.0.5 according to Tukey’s test. Data are mean ± SD.

The results presented in (Table 3) indicate that the application of SCB and ZnO NPs, either individually or in combination, significantly reduced cadmium (Cd) accumulation in the soil, roots, shoots, and seeds. Notably, the application of SCB alone was more effective in decreasing Cd levels in both soil and plant tissues compared to the use of ZnO NPs. Specifically, applying SCB at rates of 5 and 10 tons ha^−1^ resulted in reductions in Cd accumulation as follows: in soil (20.46–34.56%), roots (37.33–53.68%), shoots (33.98–58.48%), and seeds (27.31–50.53%), when compared to the treatment with 0 tons of SCB ha^−1^. Additionally, the foliar application of ZnO NPs at concentrations of 12.5 and 25 mg L^−1^ led to reductions in Cd accumulation in the soil (3.65–3.34%), roots (11.81–12.64%), shoots (30.23–36.05%), and seeds (26.38–23.25%) relative to the control treatment that contained 0 mg L^−1^ ZnO NPs. Figure 3 further illustrates that the co-application of SCB and ZnO NPs did not show a significant interaction with Cd levels in the soil or roots; however, there was a marked interaction observed in the shoots and seeds. Specifically, the combination of SCB at 10 tons ha^−1^ with ZnO NPs at 12.5 mg L^−1^ resulted in a significant reduction in Cd accumulation in seeds, achieving a decrease of 62.96%. Moreover, the application of SCB at 10 tons ha^−1^ combined with ZnO NPs at 25 mg L^−1^ led to an even more substantial reduction in Cd accumulation in the shoots, specifically 74.09%, compared to the treatment involving 0 tons of SCB ha^−1^ and 0 mg L^−1^ ZnO NPs.

### 2.4. Cd Translocation, Uptake, and Accumulation

**Figure 4:**
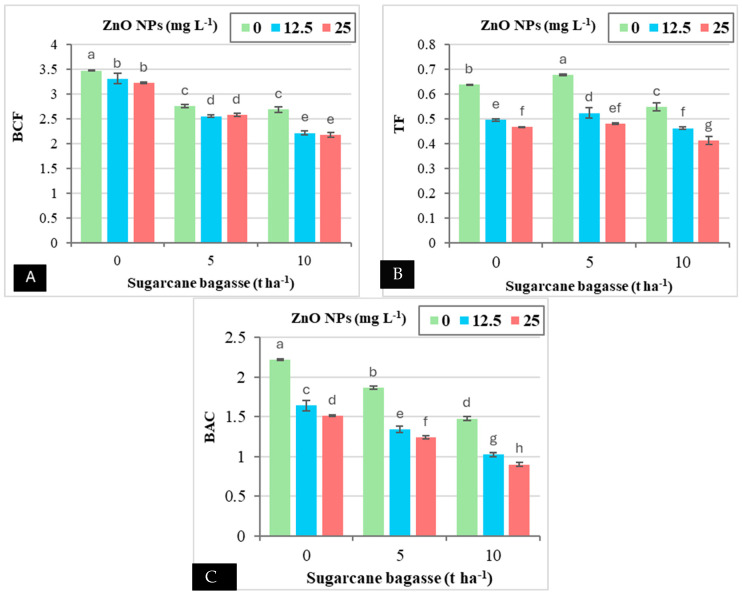
The interaction effect between sugarcane bagasse (SCB) (t ha^−1^) and zinc oxide nanoparticles (ZnO NPs) (mg L^−1^) on bioconcentration factor (BCF) (**A**), translocation factor (TF) (**B**), and bioaccumulation coefficient (BAC) (**C**). Different letters on bars show significant differences at the level of *p* ≤ 0.0.5 according to Tukey’s test. Data are mean ± SD.

The results presented in Table 4 indicate that the application of SCB and ZnO NPs altered Cd accumulation in various tissues of wheat plants compared to untreated plants, leading to reductions in bioaccumulation factor (BCF), translocation factor (TF), and bioconcentration factor (BAC). Notably, the foliar application of ZnO NPs had a more significant effect on reducing the TF compared to the application of SCB. Specifically, the application of SCB at rates of 5 and 10 tons ha^−1^ resulted in decreases in BCF (21.25–29.33%), TF (5.04–11.02%), and BAC (17.27–36.68%) when compared to the treatment with 0 tons of SCB ha^−1^. Similarly, the foliar application of ZnO NPs at concentrations of 12.5 and 25 mg L^−1^ led to reductions in BCF (9.44–10.54%), TF (20.47–26.90%), and BAC (27.97–34.38%) relative to the control treatment with 0 mg L^−1^ ZnO NPs in Cd-contaminated saline soils. Figure 4 illustrates that the combined application of SCB and ZnO NPs exhibited a significant interaction concerning BCF, TF, and BAC in wheat plants grown in Cd-contaminated saline soils (Figure 4A–C). The application of SCB at 10 tons ha^−1^, in combination with ZnO NPs at 25 mg L^−1^, resulted in substantial reductions in BCF (37.41%), TF (35.17%), and BAC (59.40%) compared to the treatment with 0 tons of SCB ha^−1^ and 0 mg L^−1^ ZnO NPs.

### 2.5. Nutrient Uptake in Leaves

Although Cd-contaminated saline soils negatively impact the uptake of K, Mg, and Zn in wheat plants, the application of SCB and ZnO NPs can enhance the absorption of these essential nutrients even under such adverse conditions, as shown in Table 5. It was observed that the application of SCB alone significantly reduced Na uptake, while simultaneously enhancing the uptake of K, Mg, and Zn in the leaves when compared to the sole application of ZnO NPs. Specifically, applying SCB at rates of 5 and 10 tons per hectare resulted in reductions in Na uptake (29.75–50.93%) and increases in the uptake of K (89.95–188.17%), Mg (42.61–84.67%), and Zn (46.75–101.17%) relative to the control treatment with 0 tons of SCB. The foliar application of ZnO NPs at concentrations of 12.5 and 25 mg L^−1^ also led to reductions in Na uptake (26.26–27.82%), while increasing the uptake of K (65.33–67.43%), Mg (30.86–32.03%), and Zn (62.44–66.83%) in comparison to the treatment with 0 mg L^−1^ ZnO NPs.

**Figure 5:**
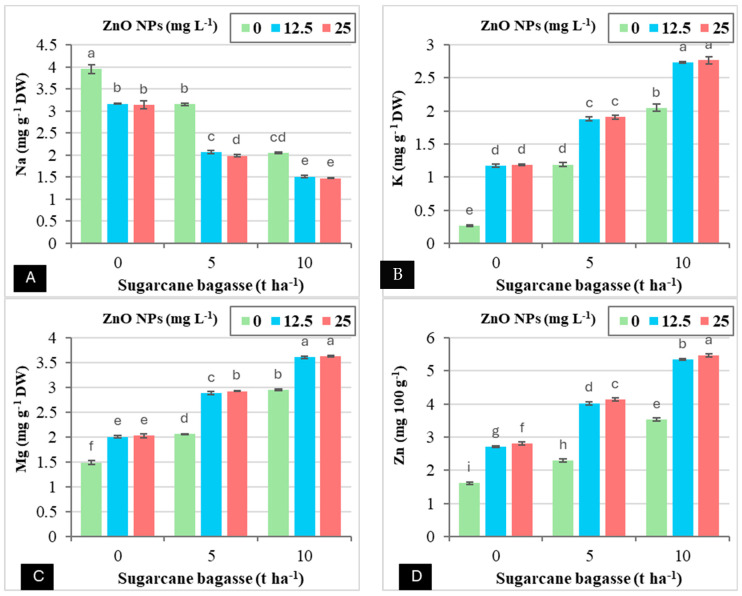
The interaction effect between sugarcane bagasse (SCB) (t ha^−1^) and zinc oxide nanoparticles (ZnO NPs) (mg L^−1^) on Na (mg g^−1^ DW) (**A**), K (mg g^−1^ DW) (**B**), Mg (mg g^−1^ DW) (**C**), and Zn (mg 100 g^−1^) (**D**). Different letters on bars show significant differences at the level of *p* ≤ 0.0.5 according to Tukey’s test. Data are mean ± SD.

Similarly, Figure 5 indicates that the combined application of SCB and ZnO NPs demonstrated a significant interaction concerning Na, K, Mg, and Zn accumulation in wheat plants grown in Cd-contaminated saline soils (Figure 5A–D). Notably, the application of SCB at 10 tons ha^−1^ combined with ZnO NPs at 25 mg L^−1^ resulted in a substantial reduction in Na accumulation (62.65%), while enhancing the accumulation of K (951.90%), Mg (143.85%), and Zn (239.26%) in comparison to the treatment with 0 tons of SCB and 0 mg L^−1^ ZnO NPs.

### 2.6. Antioxidant Enzymes Assay and Oxidative Stress

As shown in Table 6, the application of SCB, ZnO NPs, or their combination significantly increased CAT and APX levels, while simultaneously reducing MDA and H_2_O_2_ contents in leaves under Cd-contaminated saline soil. The data indicate that SCB alone resulted in a more substantial increase in CAT and APX levels, along with a greater reduction in MDA and H_2_O_2_ compared to the application of ZnO NPs alone. Applying SCB at 5 and 10 tons ha^−1^ increased CAT levels by 18.02–35.28% and APX levels by 28.25–54.96%, while simultaneously reducing MDA by 26.19–55.05% and H_2_O_2_ by 31.29–55.77% compared to SCB at 0 tons ha^−1^.

In contrast, the foliar application of ZnO NPs at concentrations of 12.5 and 25 mg L^−1^ increased CAT by 15.78–17.30% and APX by 17.25–20.13%, while it reduced MDA by 27.80–30.06% and H_2_O_2_ by 30.09–34.47% compared to ZnO NPs at 0 mg ha^−1^.

**Figure 6:**
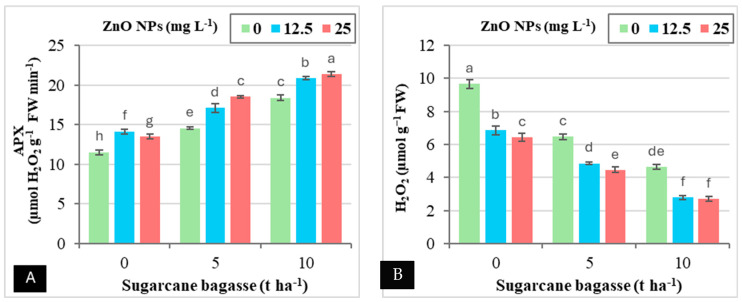
The interaction effect between sugarcane bagasse (SCB) (t ha^−1^) and zinc oxide nanoparticles (ZnO NPs) foliar application (mg L^−1^) on peroxidase (APX) (µmol H_2_O_2_ g^−1^ FW min^−1^) (**A**) and H_2_O_2_ (µmol g^−1^ FW) (**B**). Different letters on bars show significant differences at the level of *p* ≤ 0.0.5 according to Tukey’s test. Data are mean ± SD.

Figure 6A,B shows that the combined application of SCB and ZnO NPs did not result in a significant interaction for CAT or MDA levels, but there was a clear interaction with APX and H_2_O_2_ levels. Specifically, applying SCB at 10 tons ha^−1^ combined with ZnO NPs at 25 mg L^−1^ resulted in a significant increase in APX content (85.83%) and a reduction in H_2_O_2_ content (71.90%) compared to the control treatment.

### 2.7. Plant Physiological Activity

Cd-contaminated saline soils adversely affect physiological processes in plants, leading to disturbances in membrane leakage, leaf greenness (as measured by SPAD), the photosynthetic rate, stomatal conductance, and proline content in leaves (Table 7). However, the application of SCB and ZnO NPs, individually or in combination, significantly inhibited membrane leakage and proline levels, while it enhanced leaf greenness, the photosynthetic rate, and stomatal conductance. As indicated in Table 7, the application of SCB alone was more effective in reducing membrane leakage and proline levels, as well as improving leaf greenness, the photosynthetic rate, and stomatal conductance compared to the single application of ZnO NPs.

When comparing treatments, the application of SCB at 5 and 10 tons ha^−1^ resulted in reductions in membrane leakage (20.24–42.62%) and proline content (32.39–60.27%), while it increased leaf greenness (29.77–55.10%), the photosynthetic rate (35.93–79.30%), and stomatal conductance (26.11–52.65%) relative to the control treatment with 0 tons of SCB. Similarly, the foliar application of ZnO NPs at concentrations of 12.5 and 25 mg L^−1^ led to reductions in membrane leakage (16.47–19.10%) and proline levels (27.27–31.65%), while it enhanced leaf greenness (19.92–22.31%), the photosynthetic rate (28.23–30.74%), and stomatal conductance (17.99–20.06%) compared to the control with 0 mg L^−1^ ZnO NPs.

**Figure 7:**
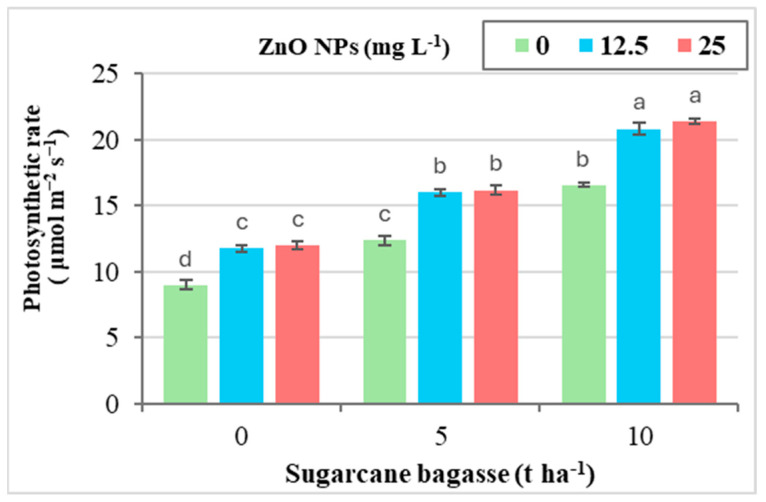
The interaction effect between sugarcane bagasse (SCB) (t ha^−1^) and zinc oxide nanoparticles (ZnO NPs) (mg L^−1^) on photosynthetic rate ( μmol m^−2^ s^−1^). Different letters on bars show significant differences at the level of *p* ≤ 0.0.5 according to Tukey’s test. Data are mean ± SD.

Figure 7 indicates that the combined application of SCB and ZnO NPs showed no significant interaction effect on membrane leakage, leaf greenness, stomatal conductance, or proline content in leaves, with the exception of the photosynthetic rate, which exhibited clear interaction between the treatments (Figure 7). Notably, the application of SCB at 10 tons ha^−1^ in combination with ZnO NPs at 25 mg L^−1^ resulted in a remarkable increase in the photosynthetic rate (137.72%) when compared to the control treatment.

### 2.8. Transcriptional Assay of Antioxidant

The application of SCB and ZnO NPs significantly downregulated the gene expression of catalase (CAT), ascorbate peroxidase (APX), and manganese superoxide dismutase (Mn-SOD) in wheat plants subjected to Cd-contaminated saline soils (Table 8). The results indicate that the application of SCB had a more pronounced effect on reducing the gene expression levels of CAT, APX, and Mn-SOD compared to the foliar application of ZnO NPs. Specifically, the application of SCB at rates of 5 and 10 tons ha^−1^ resulted in downregulations in the gene expression of CAT (32.39–60.27%), APX (20.24–42.62%), and Mn-SOD (26.19–55.05%), respectively, when compared to the treatment with 0 tons of SCB.

Similarly, the foliar application of ZnO NPs at concentrations of 12.5 and 25 mg L^−1^ led to reductions in the gene expression of CAT (27.27–31.65%), APX (16.47–19.10%), and Mn-SOD (27.80–30.06%), respectively, relative to the control with 0 mg L^−1^ ZnO NPs. Notably, the application of SCB at 10 tons ha^−1^ had a more significant effect on downregulating the gene expression of CAT, APX, and Mn-SOD in wheat plants under Cd-contaminated saline soils compared to other treatments, including the control.

### 2.9. Crop Yield Indicators

The exposure of wheat plants to Cd-contaminated saline soil resulted in a decline in yield-related traits, including the number of grains per spike, 1000-grain weight, and overall grain yield. However, the application of SCB and ZnO NPs, either individually or in combination, significantly enhanced these traits in wheat plants grown in Cd-contaminated saline soil (Table 9). The findings presented in Table 9 indicate that the application of SCB led to significant improvements in the number of grains per spike, 1000-grain weight, and total grain yield compared to the sole application of ZnO NPs.

Compared to the treatment with 0 tons of SCB ha^−1^, the applications of SCB at rates of 5 and 10 tons ha^−1^ resulted in increases in grains per spike (15.33–29.58%), 1000-grain weight (14.49–26.02%), and grain yield (22.11–42.02%), respectively. Additionally, the foliar application of ZnO NPs at concentrations of 12.5 and 25 mg L^−1^ significantly enhanced the number of grains per spike (9.81–10.53%), 1000-grain weight (7.59–8.10%), and grain yield (16.82–17.70%) when compared to the control with 0 mg L^−1^ ZnO NPs.

**Figure 8:**
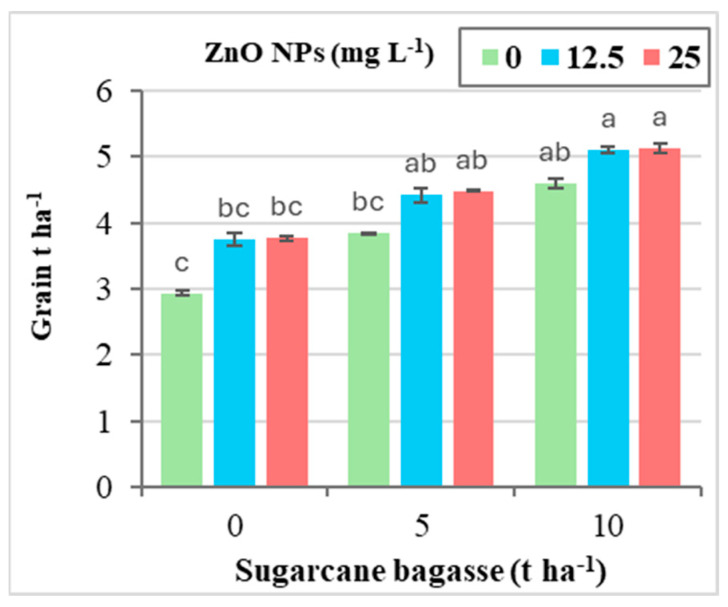
The interaction effect between sugarcane bagasse (SCB) (t ha^−1^) and zinc oxide nanoparticles (ZnO NPs) (mg L^−1^) on grain yield (ton ha^−1^). Different letters on bars show significant differences at the level of *p* ≤ 0.0.5 according to Tukey’s test. Data are mean ± SD.

Figure 8 illustrates that the combined application of SCB and ZnO NPs did not significantly affect the 1000-grain weight, but there was a notable interaction with the grain yield. Specifically, the application of SCB at 10 tons ha^−1^ in combination with ZnO NPs at 25 mg L^−1^ resulted in a significant increase in grain yield (75.18%) compared to the control treatment.

## 3. Discussion

### 3.1. Growth Inhibition in Soil Affected by Salinity and Cd

Limited information is available on the use of SCB-amended soil co-applied with ZnO NPs to mitigate the effects of salinity in co-contaminated soils. Soil salinity, when combined with heavy metal contamination, such as Cd, can severely impair plant photosynthetic activity due to oxidative stress caused by excessive ROS production. This stress leads to a reduction in antioxidant levels, ultimately hindering plant growth and productivity [32]. In our study, untreated control plots exhibited degraded soil quality, which was reflected in declines across the physical, chemical, and biological properties. This degradation resulted in the increased absorption and accumulation of Na and Cd in various plant tissues, leading to impaired physiological and biochemical processes in wheat plants. As a result, plant growth and productivity were significantly reduced, consistent with findings by [3,33].

### 3.2. SCB and ZnO NPs Enhanced Soil Characteristics Affected by Salinity and Cd

This study demonstrates improvements in the chemical, physical, and biological properties of soil when SCB-amended soil is co-applied with ZnO NPs as a pollutant barrier compared to singular treatments or the control. These enhancements are linked to the combined amendments’ effectiveness in reducing Cd content and ESP in saline and Cd-contaminated soils. Additionally, increased soil enzyme activity and microbial activity suggest enhanced nutrient availability for microorganisms in SCB-amended soils treated with ZnO NPs. These findings align with previous studies, which show that organic amendments, like biochar, improve soil health, enhance nutrient availability, and play a crucial role in maintaining cell wall and membrane integrity [34].

The increase in microbial activity also contributes to increased nitrogen fixation in the soil, promoting plant growth and development [35]. SCB biochar lowered soil pH, increased surface area, improved porosity, and enhanced soil aggregation, aeration, and permeability. These changes, in turn, enhanced drainage, reduced Na levels, lowered soil salinity, and improved physical soil properties, leading to better overall soil quality [36]. SCB significantly improved CEC and hydraulic conductivity (HC), thereby enhancing nutrient availability in the soil [19]. Furthermore, the increased microbial activity, indicated by CO_2_ influx and microbial biomass carbon (MBC), along with elevated enzymatic activity (e.g., dehydrogenase and alkaline phosphatase), led to a rise in beneficial soil microbes (e.g., *bacteria*, *Azotobacter*, and *Bacillus*). These microbes secrete polysaccharides that improve soil organic matter and quality, contributing to Cd immobilization [15,17,37].

The results showed that soil productivity in SCB-treated soils was higher than control, effectively improving soil health under saline and Cd-contaminated conditions. Soil pH, a key indicator of soil quality, decreased from moderately alkaline (8.0–8.5) to slightly alkaline (7.5–7.9) in SCB-amended soil. This change highlights SCB’s role in neutralizing soil pH and improving physicochemical properties, while also reducing Cd and Na ion content in the soil solution [38,39]. These findings are consistent with other studies that show that biochar can immobilize Cd through its negatively charged surface functional groups [40]. Moreover, biochar derived from woody materials has been found to significantly reduce Cd content within 60 days [41].

In untreated plots, high Cd levels degraded soil quality, hindering seedling growth due to a decline in biological properties, such as lower MBC, reduced microbial counts (e.g., *bacteria*, *Azotobacter*, and *Bacillus*), and decreased enzymatic activity (e.g., dehydrogenase and alkaline phosphatase), in line with other findings [42]. In contrast, SCB-amended soils, with their high carbon content, increased organic carbon levels, thereby enhancing biological and enzymatic activity [43]. An inverse relationship was observed between organic carbon content and Cd levels in contaminated soils. SCB amendments also improved soil aeration and oxygen supply, lowered soil pH, enhanced soil chemical properties, and facilitated nutrient availability, as reported in previous research [44].

It was also observed that salinity increased the mobility and toxicity of Cd in contaminated soils, making it more available for plant absorption and negatively affecting plant health. Thus, the study focused on reducing soil salinity, which is directly linked to a decrease in Cd levels in contaminated soils. SCB biochar, produced at high temperatures (400–600 °C), has a large surface area and high cation exchange capacity, in which contributed to reduced electrical conductivity (EC) and ESP. The negative charges on SCB’s functional groups restricted Na ions in the rhizosphere, thereby decreasing Cd availability by promoting its adsorption onto colloidal surfaces, away from the soil solution [15,45,46].

Zinc is an essential micronutrient for plants, required in small amounts [27], but it plays a critical role in vital processes, such as enzyme formation, root growth regulation, and maintaining leaf health [28]. One key indicator of a plant’s ability to thrive in saline and Cd-contaminated soils is chlorophyll concentration in the leaves. In this study, the application of spraying ZnO NPs promoted the accumulation of Cd and Na in the leaf cell walls, while reducing their presence in the organelles. Similarly, in the roots, Cd was concentrated in the cell walls, while its soluble portion in the root was decreased [25]. The high concentration of negatively charged active groups in the cell wall was found to bind and immobilize both Cd and Na, limiting their transport within the plant. Moreover, an antagonistic relationship between zinc and both Cd and Na was observed [26], suggesting that increasing Zn levels through spraying can reduce Cd and Na concentrations in plant tissues. For this reason, Zn was applied as NPs in our study, taking advantage of their nanoscale size and larger surface area for more efficient use [29].

Zn O NPs, ranging from 1 to 100 nm, have been shown to mitigate the harmful effects of abiotic stress on plants [29]. Therefore, using ZnO NPs instead of traditional Zn fertilizers can enhance Zn availability to meet the nutritional needs of wheat crop, while also reducing Cd and Na uptake [47]. The application of ZnO NPs in this form helped retain moisture in both leaves and roots, facilitated nutrient absorption, and improved the canopy size by enhancing photosynthetic efficiency, chlorophyll content, gas exchange, and stomatal conductance. As a result, there was an increase in the translocation of photoassimilates into grains under saline and Cd-contaminated soils [48]. Zn also plays a crucial role in respiration by aiding in the formation of respiration-related enzymes [27]. Additionally, our study revealed that Zn slightly improved the soil quality around the plant rhizosphere. However, its combined use with SCB-amended soil had a much greater impact than when applied alone. These findings align with previous studies, highlighting the important role of ex-ZnO NPs, supporting the results of our current research [30].

It has been shown that ZnO NPs are absorbed through the leaf epidermis or via endocytosis and are transported through apoplast pathways into the phloem, where they accumulate in the root cell walls and influence the rhizosphere [31]. This process enhances nutrient uptake by root cells, while decreasing Cd and Na absorption, thereby promoting seedling growth. Zn is essential for plant elongation and growth, acting as a cofactor for enzymes involved in the synthesis of tryptophan, which in turn produces the hormone auxin, crucial for plant growth and elongation [49]. Thus, the combined application of SCB and ZnO NPs are highly recommended to improve soil health, particularly by enhancing soil physicochemical and biological properties, as well as enzymatic activity in saline and Cd-contaminated soils [50].

Recent research has confirmed that ZnO NPs can enhance the absorption of essential nutrients for plant growth and metabolism, even under adverse soil conditions that would otherwise impair nutrient uptake [51]. For example, studies such as [25] have shown that the application of SCB, which is rich in carbon and nitrogen, immobilizes Cd and binds Na ions, converting them into insoluble forms in the soil solution. This reduces their uptake by roots and limits their translocation through the xylem to plant tissues, ultimately lowering BCF, TF, and BAC in saline and Cd-contaminated soils. These findings suggest that SCB-amended soil, rich in organic matter, is more effective at immobilizing Cd and binding Na compared to saline soil spiked with Cd and without SCB. Furthermore, ZnO NPs significantly reduced Cd absorption in plants by enhancing antioxidant activity and reducing free radical levels [52], consistent with their effects on antioxidant genes (CAT, APX, and Mn-SOD).

In addition, ZnO NPs reduced Cd toxicity in plants by minimizing oxidative damage, thereby improving the photosynthetic system as well as various physiological and biochemical processes. The increase in the photosynthetic rate could be linked to higher Mg levels, which are essential for chlorophyll synthesis [53]. Studies have also shown that ZnO NPs decreased Cd accumulation in different plant parts, while improving cell membrane stability [31]. Moreover, ZnO NPs enhanced wheat crop health by reducing Cd toxicity and its absorption by 30–77%, while simultaneously increasing Zn uptake [49]. This resulted in improved plant health and food safety under saline and Cd-contaminated conditions [50]. Therefore, the combined application of SCB with ZnO NPs is strongly recommended to enhance soil microbial and enzymatic activity. These findings suggest that nutrient accumulation occurs through continuous root absorption via the xylem and remobilization through the phloem from vegetative parts to grains, ultimately leading to increased crop yield and improved traits [25].

### 3.3. SCB and ZnO NPs Enhanced Physiological, Biochemical, and Wheat Yield-Related Traits Grown in Saline-Affected and Cd-Contaminated Soils

The aim of this study is to reduce the absorption, transport, and accumulation of Cd and Na ions in wheat plants, while enhancing antioxidant enzyme activity and nutrient uptake. Along with improving physiological and biochemical processes, the goal is to not only enhance soil health but also to boost plant health and crop productivity in saline and Cd-contaminated soils. Our findings showed that co-applying SCB-amended soil with ZnO NPs, compared to singular treatments or the control, significantly reduced Cd and Na uptake and accumulation in wheat plants grown in saline and Cd-contaminated soils [15]. This co-application also led to increased antioxidant enzymes activity (CAT and APX), which corresponded with the enhanced expression of antioxidant genes (CAT, APX, and Mn-SOD), as well as a decrease in oxidative stress indicators (MDA, H_2_O_2_, and EL). These improvements ultimately resulted in better nutrient uptake (K, Mg, and Zn) and improved gas exchange parameters (SPAD, Pn, gs, and Ci) in wheat plants [54]. These results are consistent with recent studies showing that SCB improves nutrient availability in the rhizosphere, enhances root absorption, facilitates nutrient transport through xylem vessels, and promotes the accumulation of key nutrients, like K and Mg, in leaves, while reducing Na levels. This improvement positively impacts photosynthesis, stomatal conductance, and CO_2_ concentration by increasing chlorophyll levels (SPAD value), leading to enhanced plant growth and development compared to untreated plants in saline and Cd-contaminated soils [54].

The study also revealed a strong relationship between SCB application and reduced Cd and Na absorption by the roots, as well as their subsequent accumulation in the leaves under these conditions [15,22]. The increased K and Mg content in the leaves, observed with SCB treatment, plays a critical role in increasing plant resilience to environmental stressors, as noted in previous research [16]. In untreated soils, higher oxidative damage was evident, with increased MDA and H_2_O_2_ levels, leading to elevated EL, the production of ROS, and damage to the cell membrane in wheat plants grown in saline and Cd-contaminated soils [18]. Conversely, SCB treatment significantly reduced oxidative stress indicators, which corresponded with enhanced antioxidant activity (CAT and APX), suggesting an improved defense system in plants under these stressful conditions. This was also associated with reduced EL and better maintenance of cell membrane integrity [19,55], consistent with findings on antioxidant gene expression (CAT, APX, and Mn-SOD) [56]. SCB helped neutralize ROS within plant cells by converting H_2_O_2_ into H_2_O and O_2_, thereby reducing protein and lipid degradation [20,21] and contributing to improved plant performance in growth and productivity.

Chlorophyll concentration in leaves is a key indicator of a plant’s ability to thrive in saline and Cd-contaminated soils [57]. The increased chlorophyll levels observed in this study were likely due to an enhanced defense system and reduced oxidative damage [58]. Additionally, ZnO NPs boosted antioxidant enzyme activities, reduced ROS levels, and improved cell membrane integrity by lowering EL [29]. These improvements were accompanied by increased nutrient uptake (K, Mg, and Zn) under saline and Cd-contaminated conditions [48]. A clear enhancement in wheat’s physiological and biochemical traits were observed when SCB was combined with ZnO NPs, as compared to individual treatments. This was reflected in grain yield and yield-related traits under these challenging soil conditions [17,47]. Our results showed that the highest efficiency in improving both soil properties and plant performance was achieved with the combined application of SCB-amended soil and ZnO NPs, as compared to individual applications of SCB or foliar spraying with ZnO NPs [30,31]. These findings suggest that nutrient accumulation in the plant occurs through continuous root absorption via the xylem and remobilization through the phloem, ultimately enhancing crop yield and yield-related traits, especially under semi-arid conditions with saline co-contaminated soils [44].

## 4. Materials and Methods

### 4.1. Experimental Site and Description

The research was conducted in an agricultural field located in the Mahalla Al-Kubra area, Gharbia Governorate, near the Kitchener Drain (latitude: 30°58.24′ N; longitude: 31°10.01′ E). During the wheat growing season from November 2022 to April 2023, the recorded weather conditions indicated an average maximum temperature of 27.22 °C and a minimum of 18.32 °C in November, with a rainfall rate of 0.48 mm and 35.77% relative humidity. By April 2023, the average maximum temperature was 25.69 °C, the minimum was 15.56 °C, rainfall was 0.51 mm, and relative humidity increased to 46.38%.

Random soil samples were collected for initial chemical and physical analysis (Table 10).

Water samples from the Kitchener Drain, the main irrigation source, were analyzed prior to the experiment. The water had a pH of 7.03 and an EC of 0.48 dS m^−1^. Concentrations of Na, Cl, NH₄, and SO₄ were 198 mg L^−1^, 3.97 mg L^−1^, 1.62 mg L^−1^, and 0.09 mg L^−1^, respectively. The Cd content was 0.097 mg L^−1^, which exceeds the acceptable irrigation limit of 0.01 mg L^−1^.

Before the experiment, the soil was plowed twice to loosen the upper layer and ensure uniform distribution of treatments. Fertilizer applications of nitrogen (N), phosphorus (P), and potassium (K) were made according to the recommendations from the Egyptian Ministry of Agriculture. The experimental area was divided into plots measuring 2 × 2.5 m, with treatments arranged in a completely randomized block design and replicated three times. Nine different treatments were applied in each replication: control (untreated), SCB at 5 t ha^−1^, SCB at 10 t ha^−1^, ZnO NPs at 12.5 mg L^−1^, ZnO NPs at 25 mg L^−1^, SCB at 5 t ha^−1^ + ZnO NPs at 12.5 mg L^−1^, SCB at 5 t ha^−1^ + ZnO NPs at 25 mg L^−1^, SCB at 10 t ha^−1^ + ZnO NPs at 12.5 mg L^−1^, and SCB at 10 t ha^−1^ + ZnO NPs at 25 mg L^−1^.

Wheat seeds (*Triticum aestivum* L., Sakha 95) were sown at a rate of 120 kg per hectare, in rows with 20 cm spaced. Irrigation was performed five times at one-month intervals, from planting to maturity, using water from the Kitchener Drain. Standard agricultural practices were followed to promote healthy plant growth throughout the wheat’s development.

### 4.2. Production and Analysis of Sugarcane SCB

Bagasse was sourced from a sugarcane processing factory in southern Egypt. After being air-dried at room temperature (25 °C), it was ground into small pieces, approximately 2 mm in size. The ground bagasse was then subjected to slow pyrolysis at 500 °C in a furnace for two hours to produce biochar. The resulting biochar was stored in containers until it was applied as a soil amendment at a rate of 10 tons per hectare, added after the second plowing and before the third.

Before applying the SCB biochar in the field experiment, samples were analyzed to determine their properties as a soil amendment, as described by [59]. The analysis revealed that the bagasse is rich in essential minerals, including nitrogen (5.11 g kg^−1^), phosphorus (6.02 g kg^−1^), potassium (198.36 g kg^−1^), zinc (91.3 g kg^−1^), calcium (31.02 g kg^−1^), and magnesium (7.98 g kg^−1^). The organic matter content was 26.58%, with carbon (C) at 527 g kg^−1^, cation exchange capacity (CEC) at 4.99 cmolc kg^−1^, and a surface area of 9.89 m^2^ g^−1^. The biochar had an electrical conductivity (EC) of 0.99 dS m^−1^, a pH of 9.2, and an extremely low Cd content (0.01 mg kg^−1^).

### 4.3. Production and Properties of ZnO NPs

In this study, ZnO NPs with a high purity of 99.9% and a particle size of 50 ± 10 nm were sourced from Sigma-Aldrich, Darmstadt, Germany, and synthesized using a chemical method. The process involved dissolving 0.5 moles of Zn acetate and 1 mole of sodium hydroxide in separate containers and shaking them for 15 min at 60 °C. The heated zinc acetate solution was then added dropwise to the hot sodium hydroxide solution, and the mixture was kept at 60 °C for 120 min. The resulting ZnO nanoparticles were thoroughly washed with sterile deionized water and homogenized at 60 °C using ultrasonic waves [60]. The ZnO NPs were applied by spraying the plants three times at 15-day intervals, with concentrations of 0, 12.5, and 25 mg L^−1^, starting 30 days after planting. Spraying was performed uniformly across the plants, with distilled water used for the control treatment.

### 4.4. Soil Analyses

To evaluate the physical and chemical properties of the soil at harvest, soil samples were collected using an auger at a depth of 0–20 cm. The soil pH was measured after the soil and foliar treatments during the growth stage by preparing a water–soil suspension (1:2.25). Additionally, a soil paste extract was prepared a separate sample to determine the electrical conductivity using a pH meter (Jenway 3510, Cambridgeshire, UK) and an EC meter (Jenway 4310, Cambridgeshire, UK), respectively [61]. The ESP was calculated using the following equation:

ESP = 1.95 + 1.03 × SAR (R^2^ = 0.92)

whereas

$$SAR=\left[Na^{+}\right]/\sqrt{\frac{\left(\left[C{a^{2}}^{+}\right]+\left[M{g^{2}}^{+}\right]\right)}{2}}$$

where Na^+^, Ca^2+^, and Mg^2+^ were expressed in meq L^−1^ Seilsepour, Rashidi [62].

Soil biological activity was assessed 80 days after sowing by collecting soil samples to measure the CO_2_ influx (mg CO_2_ 100 g^−1^ soil 24 h^−1^) and microbial biomass carbon (SMBc; mg g^−1^ soil), following the methods outlined by [63,64], respectively, based on the fumigation incubation–extraction methodology. Measuring microbial biomass carbon in soil is important, because it provides an indicator of the size of the microbial community and its role in nutrient cycling and organic matter dynamics [65]. Additionally, soil enzyme activities were evaluated at 80 days after sowing by obtaining soil samples to measure dehydrogenase and alkaline phosphatase activities. Dehydrogenase activity in soil is important, because it serves as a key indicator of microbial oxidative activity and overall soil biological health. Alkaline phosphatase activity in soil is important, because it reflects the soil’s capacity to mineralize organic phosphorus into bioavailable forms for plant uptake [66,67]. Dehydrogenase activity was measured using a spectrophotometer (Shimadzu, Kyoto, Japan) following [68] with the 2,3,5-triphenyl tetrazolium chloride (TTC) method (3% *w*/*v*), and alkaline phosphatase activity was determined using p-nitrophenyl phosphate as a substrate with absorbance measured at 440 nm, as per [67]. Bacteriological activity was also evaluated by determining the total count of bacteria, *Azotobacter*, and *Bacillus* in soil samples at 70 days, using King’s B agar medium and modified Ashby’s media, following the procedures in [69,70,71]. Measuring *Azotobacter* and *Bacillus* in soil is important, because they are key indicators of microbial health and contribute to nutrient cycling, nitrogen fixation, and plant growth promotion [72,73]. At harvest, soil samples were collected to measure Cd content, extracted using EDTA, and quantified by atomic absorption spectrophotometry (AAS, Perkin Elmer 3300, Shelton, CT, USA) [74].

### 4.5. Cd Translocation, Uptake, and Accumulation

At physiological maturity, five wheat plants were randomly selected from each experimental plot, uprooted, and carefully cleaned. After grinding, the samples were stored in separate polyethylene bags. The samples were then processed using the Kjeldahl digestion method and placed in an oven at 150 °C for 60 min. Following this preparation, the Cd content in various parts of the wheat plant was determined using flame atomic absorption spectroscopy (AAS, Perkin Elmer 3300, Shelton, CT, USA) [75]. Cd translocation, uptake, and accumulation were subsequently calculated using the following equations [76,77,78]:

$$BCF=\frac{Cd\;in\;roots}{Cd\;in\;soil}$$

$$TF=\frac{Cd\;in\;leaves}{Cd\;in\;roots}$$

$$BAC=\frac{Cd\;in\;leaves}{Cd\;in\;soil}$$

where BCF, TF, and BAC stand for bioconcentration factor, transfer factor, and bioaccumulation factor.

### 4.6. Nutrient Uptake in Leaves

After 70 days of cultivation, leaves were randomly collected from each experimental plot and stored in bags for the drying process. The leaves were dried in an oven at 70 °C, then ground into a fine powder. The powdered samples were digested with a 1:1 mixture of HNO_3_ and HClO_4_ for 120 min at 220 °C to measure potassium (K) content using a flame photometer. Sodium (Na), magnesium (Mg), and zinc (Zn) contents were analyzed using an Atomic Absorption Spectrophotometer (AAS, Perkin Elmer 3300, Shelton, CT, USA).

### 4.7. Antioxidant Enzymes Assay and Oxidative Stress

Ten discs (1 cm^2^) of fresh wheat leaves were collected to determine catalase (CAT) enzyme activity (µmol H_2_O_2_ g^−1^ FW min^−1^), which was quantified using a UV-visible spectrophotometer at 240 nm, based on the rate of H_2_O_2_ consumption, as described by [79]. Meanwhile, ascorbate peroxidase (APX) enzyme activity (µmol H_2_O_2_ g^−1^ FW min^−1^) was assessed by monitoring the decrease in optical density at 290 nm as ascorbate was oxidized, as demonstrated by [80].

Oxidative stress indices were assessed by measuring hydrogen peroxide (H_2_O_2_; µmol g^−1^ FW), which was analyzed using a UV-160A spectrophotometer (Shimadzu, Japan) at 426 nm, as outlined by [81]. Lipid peroxidation (μmol g^−1^ FW) was quantified as malondialdehyde (MDA) at 450 nm, 532 nm, and 600 nm using a spectrophotometer, following the method described by [46]. Additionally, electrolyte leakage (EL %) was calculated using the formula: (initial conductivity/final conductivity) × 100, as detailed by [82].

### 4.8. Plant Physiological Activity

At 70 days after planting, the SPAD leaf greenness index was measured using a chlorophyll meter (SPAD Model–502, Osaka, Japan), as outlined by [83]. The photosynthetic rate (Pn; μmol m^−2^ s^−1^) and stomatal conductance (gs; mmol H_2_O m^−2^ s^−1^) were determined using an open IRGA LI-COR 6400 system (LI-6400, Li-Cor Inc., Lincoln, NE, USA), following the procedure described by [84]. Additionally, the proline content was assessed in fresh wheat leaves at 520 nm using a spectrophotometer, as detailed by [85], with results expressed as mg 100 g^−1^ FW.

### 4.9. Transcriptional Assay of Antioxidant

Real-time PCR (qPCR) analysis was performed to evaluate the expression of three antioxidant genes (CAT, APX, and Mn-SOD) in wheat plants subjected to saline soil irrigated with wastewater. Three biological replicates were selected for the isolation of both RNA and cDNA using Qiagen kits. PCR reactions and amplification conditions for the CAT, APX, and Mn-SOD genes were carried out, as described by [56]. The expression levels of these antioxidant genes were determined using the 2^−ΔΔCt^ method, with ACTIN serving as the internal reference gene [86].

### 4.10. Crop Yield

At harvest time, five plants from each plot were selected to assess yield-related traits, including the number of grains per spike and the weight of 1000 grains (g). Additionally, a 2 m^2^ area from the center of each plot was harvested to determine the grain yield at 14% moisture content.

### 4.11. Statistical Analysis

The collected data were analyzed using analysis of variance (ANOVA) procedures, as described by [87], utilizing the CoStat 6.4 Statistical Software package. When significant differences were found (*p* < 0.05), mean comparisons were performed according to the method outlined by [88].

## 5. Conclusions

The combined application of SCB and ZnO NPs has shown a significant positive impact on remediating Cd-contaminated saline soils, improving wheat crop performance by reducing Cd bioavailability, enhancing soil health, and increasing plant resilience to oxidative stress. These findings suggest that this eco-friendly approach could serve as a viable alternative to conventional soil treatments in agricultural regions affected by salinity and Cd contamination. Future research should focus on scaling these treatments for different crops and soil types, exploring their long-term effects on soil microbial communities and assessing the broader environmental impacts of nanoparticle use in agriculture.

## Figures and Tables

**Table 1:** Effect of sugarcane bagasse (SCB) and zinc oxide nanoparticles (ZnO NPs) on pH, EC (dS m^−1^), ESP (%), soil CO_2_ influx (mg CO_2_ 100 g^−1^ soil 24 h^−1^), and microbial biomass carbon (mg g^−1^ Soil) of wheat plants under Cd-contaminated saline soils.

Treatment	Con.	Soil Physicochemical Properties			Soil Microbial Indicators	
		pH	EC (ds m^−1^)	ESP (%)	Soil CO_2_ Influx (mg CO_2_ 100 g^−1^ Soil 24 h^−1^)	Microbial Biomass Content (mg g^−1^ Soil)
SCB (ton ha^−1^)	0	8.4 a ± 0.3	4.42 a ± 0.11	16.22 a ± 0.91	15.92 c ± 1.22	2.23 c ± 0.32
	5	8.15 b ± 0.21	3.9 b ± 0.18	13.28 b ± 0.62	24.74 b ± 1.29	3.97 b ± 0.12
	10	7.88 c ± 0.41	3.39 c ± 0.21	11.22 c ± 0.52	35.56 a ± 1.36	5.61 a ± 0.16
ZnO NPs (mg L^−1^)	0	8.15 a ± 0.23	3.9 a ± 0.32	13.58 a ± 0.82	24.38 b ± 1.81	3.79 b ± 0.41
	12.5	8.14 a ± 0.44	3.9 a ± 0.29	13.56 a ± 0.53	26.1 a ± 1.23	4.03 a ± 0.49
	25	8.15 a ± 0.31	3.91 a ± 0.22	13.57 a ± 0.44	25.74 a ± 1.64	4 a ± 0.48
*p* _SCB_		<0.01	<0.01	<0.01	<0.01	<0.01
*p* _ZnO NPs_		0.25	0.27	<0.01	<0.01	<0.01
*p* _SCB×ZnO NPs_		0.87	0.72	0.99	0.76	<0.05

Values present the means ± standard deviation (mean ± SD) of three replicates. Different letters indicate statistically significant differences between treatments according to Tukey’s HSD test (*p* irrigation × treatment ≤ 0.05).

**Table 2:** Effect of sugarcane bagasse (SCB) and zinc oxide nanoparticles (ZnO NPs) on dehydrogenase (mg phenol kg^−1^ day^−1^), alkaline phosphatase (mg phenol kg^−1^ day^−1^), bacteria (Log cfu g^−1^ soil), *Azotobacter* (Log cfu g^−1^ soil), and *Bacillus* (Log cfu g^−1^ soil) of wheat plants under Cd-contaminated saline soils.

Treatment	Con.	Soil Enzymes Activity		Soil Bacteriological Activity		
		Dehydrogenase (mg Phenol kg^−1^ Day^−1^)	Alkaline Phosphatase (mg Phenol kg^−1^ Day^−1^)	Bacteria (Log cfu g^−1^ Soil)	Azotobacter (Log cfu g^−1^ Soil)	Bacillus (Log cfu g^−1^ Soil)
SCB (ton ha^−1^)	0	12.35 c ± 0.43	0.29 c ± 0.021	2.64 c ± 0.26	0.95 c ± 0.17	1.9 c ± 0.28
	5	29.93 b ± 1.04	0.53 b ± 0.025	5.2 b ± 0.35	2.25 b ± 0.23	3.76 b ± 0.17
	10	44.21 a ± 0.58	0.78 a ± 0.029	7.79 a ± 0.43	4.46 a ± 0.39	5.78 a ± 0.23
ZnO NPs (mg L^−1^)	0	28.11 b ± 0.13	0.5 b ± 0.22	4.76 b ± 0.22	2.21 b ± 0.41	3.52 b ± 0.71
	12.5	29.28 a ± 0.96	0.55 a ± 0.21	5.45 a ± 0.35	2.73 a ± 0.57	3.98 a ± 0.65
	25	29.09 a ± 0.84	0.54 a ± 0.21	5.42 a ± 0.26	2.73 a ± 0.62	3.95 a ± 0.69
*p* _SCB_		<0.01	<0.01	<0.01	<0.01	<0.01
*p* _ZnO NPs_		<0.01	<0.01	<0.01	<0.01	<0.01
*p* _SCB × ZnO NPs_		0.44	0.81	<0.01	<0.01	<0.01

Values present the means ± standard deviation (mean ± SD) of three replicates. Different letters indicate statistically significant differences between treatments according to Tukey’s HSD test (*p* irrigation × treatment ≤ 0.05).

**Table 3:** Effect of sugarcane bagasse (SCB) and zinc oxide nanoparticles (ZnO NPs) on extractable soil cadmium content (mg kg^−1^), Cd content in roots (µg g^−1^), Cd content in shoots (µg g^−1^), and Cd content in seeds (µg g^−1^) of wheat plants under Cd-contaminated saline soils.

Treatment	Con.	Cd in Soil (mg kg^−1^)	Cd in Roots (µg g^−1^)	Cd in Shoots (µg g^−1^)	Cd in Seeds (µg g^−1^)
SCB (ton ha^−1^)	0	0.87 a ± 0.011	2.92 a ± 0.13	1.57 a ± 0.05	0.8 a ± 0.03
	5	0.7 b ± 0.021	1.83 b ± 0.12	1.04 b ± 0.09	0.58 b ± 0.05
	10	0.57 c ± 0.013	1.35 c ± 0.17	0.65 c ± 0.02	0.4 c ± 0.02
ZnO NPs (mg L^−1^)	0	0.73 a ± 0.056	2.22 a ± 0.67	1.39 a ± 0.08	0.71 a ± 0.02
	12.5	0.7 b ± 0.044	1.95 b ± 0.71	0.97 b ± 0.07	0.52 c ± 0.06
	25	0.71 b ± 0.035	1.94 b ± 0.7	0.89 c ± 0.05	0.54 b ± 0.05
*p* _SCB_		<0.01	<0.01	<0.01	<0.01
*p* _ZnO NPs_		<0.01	<0.01	<0.01	<0.01
*p* _SCB × ZnO NPs_		0.15	0.12	<0.01	<0.01

Values present the means ± standard deviation (mean ± SD) of three replicates. Different letters indicate statistically significant differences between treatments according to Tukey’s HSD test (*p* irrigation × treatment ≤ 0.05).

**Table 4:** Effect of sugarcane bagasse (SCB) and zinc oxide nanoparticles (ZnO NPs) on bioconcentration factor (BCF), translocation factor (TF), and bioaccumulation coefficient (BAC) of wheat plants under Cd-contaminated saline soils.

Treatment	Con.	BCF	TF	BAC
SCB (ton ha^−1^)	0	3.34 a ± 0.12	0.53 a ± 0.08	1.79 a ± 0.33
	5	2.63 b ± 0.11	0.56 a ± 0.09	1.48 b ± 0.29
	10	2.36 c ± 0.25	0.48 b ± 0.06	1.13 c ± 0.27
ZnO NPs (mg L^−1^)	0	2.98 a ± 0.38	0.62 a ± 0.06	1.85 a ± 0.32
	12.5	2.69 b ± 0.49	0.49 b ± 0.03	1.34 b ± 0.27
	25	2.66 b ± 0.46	0.45 c ± 0.04	1.22 c ± 0.27
*p* _SCB_		<0.01	<0.01	<0.01
*p* _ZnO NPs_		<0.01	<0.01	<0.01
*p* _SCB × ZnO NPs_		<0.01	<0.01	<0.01

Values present the means ± standard deviation (mean ± SD) of three replicates. Different letters indicate statistically significant differences between treatments according to Tukey’s HSD test (*p* irrigation × treatment ≤ 0.05).

**Table 5:** Effect of sugarcane bagasse (SCB) and zinc oxide nanoparticles (ZnO NPs) on Na (mg g^−1^ DW), K (mg g^−1^ DW), Mg (mg g^−1^ DW), and Zn (mg g^−1^ DW) of wheat leaves under Cd-contaminated saline soils.

Treatment	Con.	Na (mg g^−1^ DW)	K (mg g^−1^ DW)	Mg (mg g^−1^ DW)	Zn (mg g^−1^ DW)
SCB (ton ha^−1^)	0	3.42 a ± 0.13	0.87 c ± 0.03	1.84 c ± 0.16	2.38 c ± 0.18
	5	2.4 b ± 0.0.9	1.66 b ± 0.09	2.63 b ± 0.12	3.49 b ± 0.19
	10	1.68 c ± 0.06	2.52 a ± 0.15	3.4 a ± 0.16	4.79 a ± 0.28
ZnO NPs (mg L^−1^)	0	3.05 a ± 0.13	1.17 b ± 0.07	2.17 c ± 0.14	2.48 c ± 0.14
	12.5	2.25 b ± 0.07	1.93 a ± 0.06	2.84 b ± 0.11	4.03 b ± 0.28
	25	2.2 c ± 0.07	1.95 a ± 0.08	2.86 a ± 0.12	4.14 a ± 025
*p* _SCB_		<0.01	<0.01	<0.01	<0.01
*p* _ZnO NPs_		<0.01	<0.01	<0.01	<0.01
*p* _SCB × ZnO NPs_		<0.01	<0.01	<0.01	<0.01

Values present the means ± standard deviation (mean ± SD) of three replicates. Different letters indicate statistically significant differences between treatments according to Tukey’s HSD test (*p* irrigation × treatment ≤ 0.05).

**Table 6:** Effect of sugarcane bagasse (SCB) and zinc oxide nanoparticles (ZnO NPs) on catalase (CAT; µmol H_2_O_2_ g^−1^ FW min^−1^), ascorbate peroxidase (APX; µmol H_2_O_2_ g^−1^ FW min^−1^), malondialdehyde (MDA; μmol g^−1^ FW), and H_2_O_2_ (µmol g^−1^ FW) of wheat plants under Cd-contaminated saline soils.

Treatment	Con.	Antioxidant Enzymes Activity		Oxidative Stress Indicators	
		CAT (µmol H_2_O_2_ g^−1^ FW min^−1^)	APX (µmol H_2_O_2_ g^−1^ FW min^−1^)	MDA (μmol g^−1^ FW)	H_2_O_2_ (µmol g^−1^ FW)
SCB (ton ha^−1^)	0	45.3 c ± 1.58	13.05 c ± 0.21	18.24 a ± 0.62	7.66 a ± 0.53
	5	53.47 b ± 2.22	16.74 b ± 0.78	13.47 b ± 0.97	5.26 b ± 0.33
	10	61.29 a ± 2.39	20.23 a ± 0.42	8.2 c ± 0.71	3.39 c ± 0.25
ZnO NPs (mg L^−1^)	0	48.05 b ± 1.54	14.83 c ± 0.99	16.48 a ± 0.95	6.92 a ± 0.21
	12.5	55.64 a ± 2.04	17.38 b ± 1.1	11.9 b ± 0.49	4.84 b ± 0.77
	25	56.37 a ± 2.33	17.81 a ± 1.05	11.53 b ± 0.55	4.54 c ± 0.62
*p* _SCB_		<0.01	<0.01	<0.01	<0.01
*p* _ZnO NPs_		<0.01	<0.01	<0.01	<0.01
*p* _SCB × ZnO NPs_		0.55	<0.01	0.16	<0.01

Values present the means ± standard deviation (mean ± SD) of three replicates. Different letters indicate statistically significant differences between treatments according to Tukey’s HSD test (*p* irrigation × treatment ≤ 0.05).

**Table 7:** Effect of sugarcane bagasse (SCB) and zinc oxide nanoparticles (ZnO NPs) on membrane leakage (ML; %), leaf greens (SPAD), photosynthetic rate (μmol m^−2^ s^−1^), stomatal conductance (gs; mmol H_2_O m^−2^ s^−1^), and proline content (mg 100 g^−1^ FW) of wheat plants under Cd-contaminated saline soils.

Treatment	Con.	ML (%)	Leaf Greenness (SPAD)	Photosynthetic Rate (μmol m^−2^ s^−1^)	gs (mmol H_2_O m^−2^ s^−1^)	Proline (mg 100 g^−1^ FW)
SCB (ton ha^−1^)	0	36.87 a ± 1.62	28.19 c ± 1.07	10.93 c ± 0.47	35.93 c ± 1.63	11.03 a ± 0.54
	5	29.41 b ± 1.51	36.58 b ± 1.98	14.85 b ± 0.91	45.31 b ± 2.73	7.46 b ± 0.26
	10	21.16 c ± 1.14	43.72 a ± 1.29	19.59 a ± 1.02	54.84 a ± 2.39	4.38 c ± 0.47
ZnO NPs (mg L^−1^)	0	33.07 a ± 1.53	31.7 b ± 1.26	12.64 b ± 0.89	40.25 b ± 2.93	9.48 a ± 0.92
	12.5	27.62 b ± 1.32	38.01 a ± 1.65	16.21 a ± 0.93	47.5 a ± 2.01	6.9 b ± 0.35
	25	26.75 b ± 1.74	38.77 a ± 1.44	16.52 a ± 1.08	48.33 a ± 2.74	6.48 c ± 0.73
*p* _SCB_		<0.01	<0.01	<0.01	<0.01	<0.01
*p* _ZnO NPs_		<0.01	<0.01	<0.01	<0.01	<0.01
*p* _SCB × ZnO NPs_		0.29	0.11	<0.05	0.13	0.08

Values present the means ± standard deviation (mean ± SD) of three replicates. Different letters indicate statistically significant differences between treatments according to Tukey’s HSD test (*p* irrigation × treatment ≤ 0.05).

**Table 8:** Effect of sugarcane bagasse (SCB) and zinc oxide nanoparticles (ZnO NPs) on gene expression of catalase (CAT), ascorbate peroxidase (APX), and manganese superoxide dismutase (Mn-SOD) of wheat plants under Cd-contaminated saline soils.

Treatment	Con.	*CAT*	*APX*	*Mn-SOD*
SCB (ton ha^−1^)	0	3.68 a ± 0.25	3.69 a ± 0.26	2.61 a ± 0.37
	5	2.49 b ± 0.17	2.94 b ± 0.35	1.92 b ± 0.28
	10	1.46 c ± 0.10	2.12 c ± 0.31	1.17 c ± 0.39
ZnO NPs (mg L^−1^)	0	3.16 a ± 0.17	3.31 a ± 0.65	2.35 a ± 0.62
	12.5	2.3 b ± 0.12	2.76 b ± 0.73	1.7 b ± 0.64
	25	2.16 c ± 0.11	2.68 b ± 0.67	1.65 b ± 0.62
*p* _SCB_		<0.01	<0.01	<0.01
*p* _ZnO NPs_		<0.01	<0.01	<0.01
*p* _SCB × ZnO NPs_		0.09	0.29	0.15

Values present the means ± standard deviation (mean ± SD) of three replicates. Different letters indicate statistically significant differences between treatments according to Tukey’s HSD test (*p* irrigation × treatment ≤ 0.05).

**Table 9:** Effect of sugarcane bagasse (SCB) and zinc oxide nanoparticles (ZnO NPs) on number of grains per spike, 1000-grain weight (g), and grain yield (ton ha^−1^) of wheat plants under Cd-contaminated saline soils.

Treatment	Con.	Number of Grains Per Spike	1000-Grain Weight (g)	Grain Yield (ton ha^−1^)
SCB (ton ha^−1^)	0	38.15 c ± 1.61	40.54 c ± 1.39	3.48 c ± 0.42
	5	44 b ± 2.5	46.41 b ± 1.87	4.25 b ± 0.31
	10	49.43 a ± 2.35	51.09 a ± 2.01	4.95 a ± 0.27
ZnO NPs (mg L^−1^)	0	41.07 b ± 2.45	43.73 b ± 1.22	3.79 b ± 0.72
	12.5	45.1 a ± 2.11	47.05 a ± 1.74	4.43 a ± 0.59
	25	45.4 a ± 2.19	47.27 a ± 1.82	4.46 a ± 0.6
*p* _SCB_		<0.01	<0.01	<0.01
*p* _ZnO NPs_		<0.01	<0.01	<0.01
*p* _SCB × ZnO NPs_		<0.05	0.09	0.15

Values present the means ± standard deviation (mean ± SD) of three replicates. Different letters indicate statistically significant differences between treatments according to Tukey’s HSD test (*p* irrigation × treatment ≤ 0.05).

**Table 10:** Physical and chemical properties of experimental soil.

Parameter	Value
pH	8.43 ± 0.01
Electrical conductivity (EC: dS m^−1^)	4.43 ± 0.03
Soil organic matter (g kg^−1^)	10.82 ± 0.21
Exchangeable sodium percentage (ESP; %)	16.22 ± 0.42
Soluble ions (meq L^−1^)	
Ca^2+^	7.01 ± 0.08
Mg^2+^	5.73 ± 0.09
Na^+^	27.03 ± 1.21
K^+^	0.36 ± 0.01
HCO₃^−^	4.67 ± 0.05
Cl^−^	25.77 ± 1.11
SO₄^2−^	16.34 ± 1.01
Available macronutrients (mg kg^−1^)	
Nitrogen	9.87 ± 0.8
Phosphorus	7.89 ± 0.9
Potassium	367 ± 23
Cadmium content (mg kg^−1^)	
Total	5.17 ± 0.12
Extractable	0.96 ± 0.01
Texture	Clayey

## Data Availability

All data are included in the manuscript.
